# Retinal Optical Coherence Tomography Features Associated With Incident and Prevalent Parkinson Disease

**DOI:** 10.1212/WNL.0000000000207727

**Published:** 2023-10-17

**Authors:** Siegfried Karl Wagner, David Romero-Bascones, Mario Cortina-Borja, Dominic J. Williamson, Robbert R. Struyven, Yukun Zhou, Salil Patel, Rimona S. Weil, Chrystalina A. Antoniades, Eric J. Topol, Edward Korot, Paul J. Foster, Konstantinos Balaskas, Unai Ayala, Maitane Barrenechea, Iñigo Gabilondo, Anthony H.V. Schapira, Anthony P. Khawaja, Praveen J. Patel, Jugnoo S. Rahi, Alastair K. Denniston, Axel Petzold, Pearse Andrew Keane, for UK Biobank Eye & Vision Consortium

**Affiliations:** From the Institute of Ophthalmology (S.K.W., D.J.W., R.R.S., Y.Z., P.J.F., K.B., A.P.K., P.J.P., J.S.R., A.P., P.A.K.), University College London; NIHR Biomedical Research Centre at Moorfields Eye Hospital and UCL Institute of Ophthalmology (S.K.W., D.R.-B., D.J.W., R.R.S., Y.Z., E.K., P.J.F., K.B., A.P.K., P.J.P., J.S.R., A.K.D., A.P., P.A.K.), London, United Kingdom; Biomedical Engineering Department (D.R.-B., E.K., U.A., M.B.), Faculty of Engineering (MU-ENG), Mondragon Unibertsitatea, Spain; Great Ormond Street Institute of Child Health (M.C.-B., J.S.R.), and Centre for Medical Image Computing (D.J.W., R.R.S., Y.Z.), Department of Computer Science, University College London; NeuroMetrology Lab (S.P., C.A.A.), Nuffield Department of Clinical Neurosciences, University of Oxford; Dementia Research Centre (R.S.W.), University College London, United Kingdom; Department of Molecular Medicine (E.J.T.), Scripps Research, La Jolla, CA; Byers Eye Institute (E.K.), Stanford University, Palo Alto, CA; Biocruces Bizkaia Health Research Institute (I.G.), Barakaldo; IKERBASQUE: The Basque Foundation for Science (I.G.), Bilbao, Spain; Department of Clinical and Movement Neurosciences (A.H.V.S.), UCL Queen Square Institute of Neurology; Great Ormond Street Hospital NHS Foundation Trust (J.S.R.); Ulverscroft Vision Research Group (J.S.R.), University College London; NIHR Biomedical Research Centre at UCL Great Ormond Street Institute of Child Health and Great Ormond Street Hospital (J.S.R.), London; University of Birmingham (A.K.D.); University Hospitals Birmingham NHS Foundation Trust (A.K.D.); NIHR Birmingham Biomedical Research Centre (A.K.D.), University of Birmingham; and Queen Square Institute of Neurology (A.P.), University College London, United Kingdom.

## Abstract

**Background and Objectives:**

Cadaveric studies have shown disease-related neurodegeneration and other morphological abnormalities in the retina of individuals with Parkinson disease (PD); however, it remains unclear whether this can be reliably detected with in vivo imaging. We investigated inner retinal anatomy, measured using optical coherence tomography (OCT), in prevalent PD and subsequently assessed the association of these markers with the development of PD using a prospective research cohort.

**Methods:**

This cross-sectional analysis used data from 2 studies. For the detection of retinal markers in prevalent PD, we used data from AlzEye, a retrospective cohort of 154,830 patients aged 40 years and older attending secondary care ophthalmic hospitals in London, United Kingdom, between 2008 and 2018. For the evaluation of retinal markers in incident PD, we used data from UK Biobank, a prospective population-based cohort where 67,311 volunteers aged 40–69 years were recruited between 2006 and 2010 and underwent retinal imaging. Macular retinal nerve fiber layer (mRNFL), ganglion cell–inner plexiform layer (GCIPL), and inner nuclear layer (INL) thicknesses were extracted from fovea-centered OCT. Linear mixed-effects models were fitted to examine the association between prevalent PD and retinal thicknesses. Hazard ratios for the association between time to PD diagnosis and retinal thicknesses were estimated using frailty models.

**Results:**

Within the AlzEye cohort, there were 700 individuals with prevalent PD and 105,770 controls (mean age 65.5 ± 13.5 years, 51.7% female). Individuals with prevalent PD had thinner GCIPL (−2.12 μm, 95% CI −3.17 to −1.07, *p* = 8.2 × 10^−5^) and INL (−0.99 μm, 95% CI −1.52 to −0.47, *p* = 2.1 × 10^−4^). The UK Biobank included 50,405 participants (mean age 56.1 ± 8.2 years, 54.7% female), of whom 53 developed PD at a mean of 2,653 ± 851 days. Thinner GCIPL (hazard ratio [HR] 0.62 per SD increase, 95% CI 0.46–0.84, *p* = 0.002) and thinner INL (HR 0.70, 95% CI 0.51–0.96, *p* = 0.026) were also associated with incident PD.

**Discussion:**

Individuals with PD have reduced thickness of the INL and GCIPL of the retina. Involvement of these layers several years before clinical presentation highlight a potential role for retinal imaging for at-risk stratification of PD.

## Introduction

Parkinson disease (PD) is a heterogenous progressive movement disorder characterized by a loss of nigrostriatal dopaminergic neurons. Dopaminergic degeneration is detectable early with multimodal brain imaging, suggesting some striatal territories are affected decades before diagnosis.^1,2^ Individuals with prodromal PD have increased nigral iron deposition on susceptibility MRI and accelerated dopaminergic dysfunction on serial dopamine transport (DAT) scanning.^3,4^ However, brain imaging for diagnosis and disease monitoring in PD is limited as a scalable resource. DAT imaging is relatively costly, has modest availability, and requires IV contrast. MRI has shown promise for disease diagnosis and monitoring but has not yet been validated for these purposes.

Another attractive location for interrogation of dopaminergic pathology is the eye. Embryologically derived from the primitive forebrain, the retina provides a minimally invasive window into the CNS and can be imaged rapidly using modern high-resolution devices. The dopaminergic cells of the neurosensory retina are located in the inner plexiform layer (IPL) and inner nuclear layer (INL), where they mediate intercellular coupling between AII amacrine cells, horizontal cells, and retinal ganglion cells.^5,6^ Stimulated by the postmortem finding of reduced dopamine content in the retina of people with PD,^7^ researchers have sought evidence of retinal changes on in vivo imaging techniques, such as optical coherence tomography (OCT). OCT, an interferometry-based noncontact imaging modality (axial resolution approximately 5 μm), has shown diagnostic and prognostic utility in several neurologic disorders^8,9^ and is increasingly available in hospital and community settings.^10^ Studies using OCT have revealed several potential morphological abnormalities associated with PD but with inconsistency between studies. In a systematic review of 10 studies including a total of 690 participants, PD was associated with reduced thickness of the macular ganglion cell–IPL (GCIPL) and macular retinal nerve fiber layer (mRNFL). There was, however, a significant publication bias noted, and some studies did not report key details, such as the age of controls.^11^ The cell bodies of dopaminergic neurons sit at the border of INL and IPL^12^; however, the 2 studies reporting significant associations in a recent meta-analysis showed opposite directions of effect of PD with the INL.^11^ Most studies also exclude individuals with other medical comorbidities, but the natural history of PD may differ in individuals with other diseases, such as diabetes mellitus, thus limiting the external validity of these findings to the wider patient group encountered by neurologists.^13^

In this report, we leveraged a bidirectional approach analyzing retinal imaging data from individuals with PD both before and after diagnosis. Our aims were firstly, to characterize inner retinal anatomy, as measured using OCT, in individuals with prevalent PD from a large ethnically diverse real-world population study (AlzEye) and secondly to investigate the association of OCT-based measures with the development of PD using the deeply phenotyped prospective UK Biobank (UKBB) cohort. We hypothesized that individuals with prevalent PD would exhibit thinner GCIPL, mRNFL, and INL and that this difference would be associated with incident disease.

## Methods

### Design, Participants, and Setting

This cross-sectional analysis used data from the AlzEye and UKBB studies to explore retinal morphology in prevalent and incident PD, respectively. AlzEye is a retrospective cohort study, where individual-level ophthalmic data have been linked with hospital admissions across England for patients who were aged 40 years and older and had attended Moorfields Eye Hospital NHS Foundation Trust in London, United Kingdom, between January 1, 2008, and April 1, 2018. Further details about AlzEye have been published previously.^14^ UKBB is a prospective population-based multicenter cohort study of approximately 500,000 participants residing in the United Kingdom and registered with the National Health Service. Participants aged 40–69 years were initially recruited between 2006 and 2010. In addition to baseline questionnaires and physical measurements, a subset of 67,311 UKBB participants additionally underwent a detailed ophthalmic assessment including retinal imaging at their initial assessment visit. Comprehensive study protocols have been published online.

### Retinal Imaging

Nonmydriatic macula-centered OCT imaging was acquired by trained technicians from participants in both AlzEye and UKBB using Topcon imaging devices (Topcon Corp., Tokyo, Japan). In AlzEye, images were acquired using 4 different devices (3D OCT 1000, 3D OCT 1000 Mark II, 3D OCT 2000, and Triton); for UKBB, all images were acquired on the 3D OCT 1000 Mark II. All images were volume scans and covered a 6.0 mm × 6.0 mm^2^ area and had 128 horizontal B scans and 512 A scans per B scan. Images from both eyes, where available, were used. In UKBB, we only included participants who had retinal imaging acquired at the initial assessment visit (baseline instance) because this corresponded to the same time as their touchscreen questionnaire response. mRNFL, GCIPL, and INL thicknesses were estimated from OCT using the Topcon Advanced Boundary Segmentation Tool (TABS) version 1.6.2.6, a software providing automated segmentation of retinal sublayers using dual-scale gradients.^15^ Given previous evidence of parafoveal spatially relevant differences in PD and other neurodegenerative conditions, we investigated retinal sublayers for the 4 parafoveal subfields, as defined by the Early Treatment for Diabetic Retinopathy Study (ETDRS), as well as averages for the 4 inner subfields.^16^ TABS provides additional metadata for each image to establish scan quality based on segmentation error, movement artefact, and poor quality. For image quality control, we excluded the poorest 20% of images based on these specific image quality control metadata, applying the same method to both cohort data sets (further details in the Supplementary material, links.lww.com/WNL/D56).

### Systemic and Ocular Disease Variables

PD was defined using hospital admissions data from Hospital Episode Statistics (HES), a national repository of all hospital admissions in England under the provisions of the NHS (at least 97% of hospital admissions in England^17^). HES is coded using the *International Classification of Diseases, 10th Revision (ICD-10)*. PD was defined as a HES episode with *ICD-10* code G20. HES-based diagnostic codes for PD have recently been validated in a subset of 20,000 participants of UKBB and had a positive predictive value of 0.84 (95% CI 0.68–0.94).^18^ For investigating retinal markers in prevalent PD, eligible cases were defined as images after the relevant *ICD-10* code. Given previous evidence of reduced thickness of the GCIPL and mRNFL in dementia, we excluded individuals with *ICD-10* codes for all-cause dementia (E512, F00, F01, F02, F03, F106, F107, G30, G310).^19^ For defining incident PD in UKBB, we excluded those who self-reported having PD at their initial assessment visit when they had retinal imaging and then used the first hospital admission with an *ICD-10* code indicating PD as the time of disease onset. We additionally excluded those who self-reported eye disease at the initial assessment visit. Secondary exposure variables included age, sex, ethnicity, hypertension (*ICD*: I10, I15), and diabetes mellitus (*ICD*: E10, E11). Ethnicity, as self-reported by participants, was aggregated into 4 groups as defined by the UK Census (eTable 1, links.lww.com/WNL/D56). Glaucoma was defined as any patient attending the glaucoma clinic 3 or more times with ongoing follow-up as previously described.^14^ Hypertension and diabetes mellitus were defined using HES diagnostic codes for the AlzEye analysis and through self-report at the initial assessment visit touchscreen questionnaire for UKBB. Further details regarding the Data Fields used for UKBB can be found in eTable 2.

### Statistical Analysis

Initial data distributions were analyzed visually and statistically. Continuous variables were compared between groups using the Student *t* test and categorical variables through the *U* statistic permutation test of independence.^20^ To examine the association between prevalent PD and retinal morphology, we fitted linear mixed-effects models with a random intercept at the individual level to account for the multilevel structure of eyes nested in participants. Models were fitted through maximum likelihood estimation and adjusted for age, sex, ethnicity group, diabetes mellitus, and hypertension. To assess the risk of residual confounding (e.g., spuriously reduced retinal thickness because of individuals with PD having more advanced diabetic eye disease), we also performed a sensitivity analysis excluding all individuals with diabetes mellitus. Degrees of freedom were estimated using Satterthwaite's approximation.^21^ Data on self-reported ethnicity were missing for 19.4% of participants in the AlzEye cohort. Given previous evidence on the determinants of missingness of self-reported demographic data in health care, we assumed ethnicity data were missing at random.^22^ We therefore performed conditional multiple imputation with chained equations 10 times with 5 iterations using multinomial logistic regression models on all exposure and outcome variables, in their raw form, and pooled adjusted regression coefficients using Rubin's rule.^23^

To examine the association between retinal morphology and incident PD, we estimated cause-specific adjusted hazard ratios (HRs) fitting survival models including a gamma-distributed random effect on the intercept representing frailty at the individual level.^24^ The at-risk period was defined from the time of retinal imaging acquisition (the UKBB initial assessment visit data) until the earliest of death, hospital admission with a PD diagnostic code, or conclusion of the data refresh date for our UKBB application (December 1, 2020). We conducted survival analysis using a complete-case approach given the small amount of missingness for ethnicity in UKBB after image quality control (<0.3% of total). Given that previous evidence has shown HES-based codes for other neurodegenerative diseases can postdate their appearance in primary care, we additionally performed a sensitivity analysis excluding all incident cases within 24 months of retinal imaging.^25^ Statistical significance was set at *p* < 0.05. All analyses were conducted in R version 4.1.0 (R Core Team, R Foundation for Statistical Computing, Vienna, Austria; 2021) and used the mice, survival, and lmer packages.

Reporting is in line with the guidelines set by the Strengthening the Reporting of Observational Studies in Epidemiology^26^ and the recommendations of the Advised Protocol for OCT Study Terminology and Elements.^27,28^

### Standard Protocol Approvals, Registrations, and Patient Consents

The AlzEye study has received institutional and ethical review board approval, including an exemption of participant consent (REC reference 18/LO/1163). UKBB is conducted under the approvals of the North-West Research Ethics Committee (ref 06/MRE08/75); specific approval was obtained for this project (application ID 2112). All participants gave written informed consent according to the Declaration of Helsinki.

### Data Availability

National and international collaborations are welcomed; however, the data are subject to the contractual restrictions of the data sharing agreements between National Health Service Digital, Moorfields Eye Hospital, and University College London and are therefore not available for access beyond the AlzEye research team. Researchers should contact the Chief Investigator at p.keane@ucl.ac.uk. Data from the UK Biobank is available to approved researchers on application. Further information is available at ukbiobank.ac.uk/.

## Results

### Retinal Morphology in Prevalent PD

**Figure 1:**
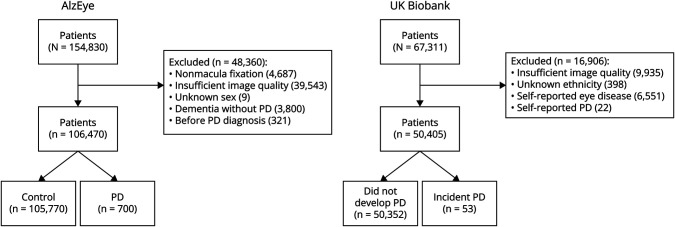
Flowchart Detailing Inclusion and Exclusion of Participants in Both AlzEye and UK Biobank PD = Parkinson disease.

**Figure 2:**
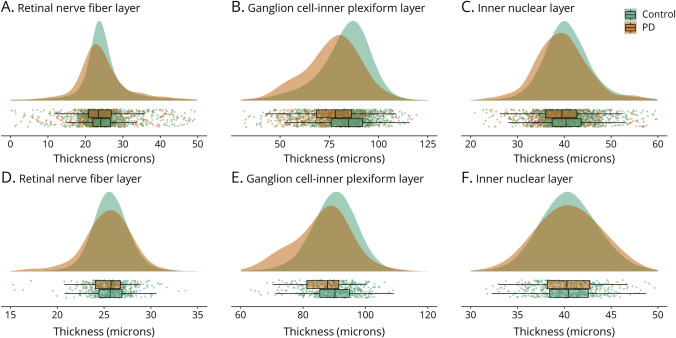
Distribution of Retinal Sublayer Thicknesses in AlzEye and UK Biobank Raincloud plots consisting of a density, box-whisker, and scatter plots for AlzEye (A–C) and UK Biobank (D–F) for individual retinal sublayers. Scatter points represent the mean of both eyes (where available) per participant. To improve visibility, a random 2% of control participants are illustrated.

From the AlzEye cohort of 154,830 with retinal imaging, there were 700 individuals (0.45%) who had prevalent PD and 105,770 controls (Figure 1). Those with PD were older, more likely to be male, hypertensive, and have diabetes mellitus (Table 1, all *p* < 0.001). In unadjusted analysis, GCIPL and INL were significantly thinner across all parafoveal locations in patients with PD compared with controls (Figure 2, all *p* < 0.001). mRNFL was also thinner in individuals with PD in all regions except the nasal subfield. Examination of those with missing ethnicity data showed that individuals, who chose not to self-report their ethnicity, were less likely to have PD. They were also younger and less likely to have hypertension, diabetes mellitus, and glaucoma (eTable 3, links.lww.com/WNL/D56).

**Table 1:**
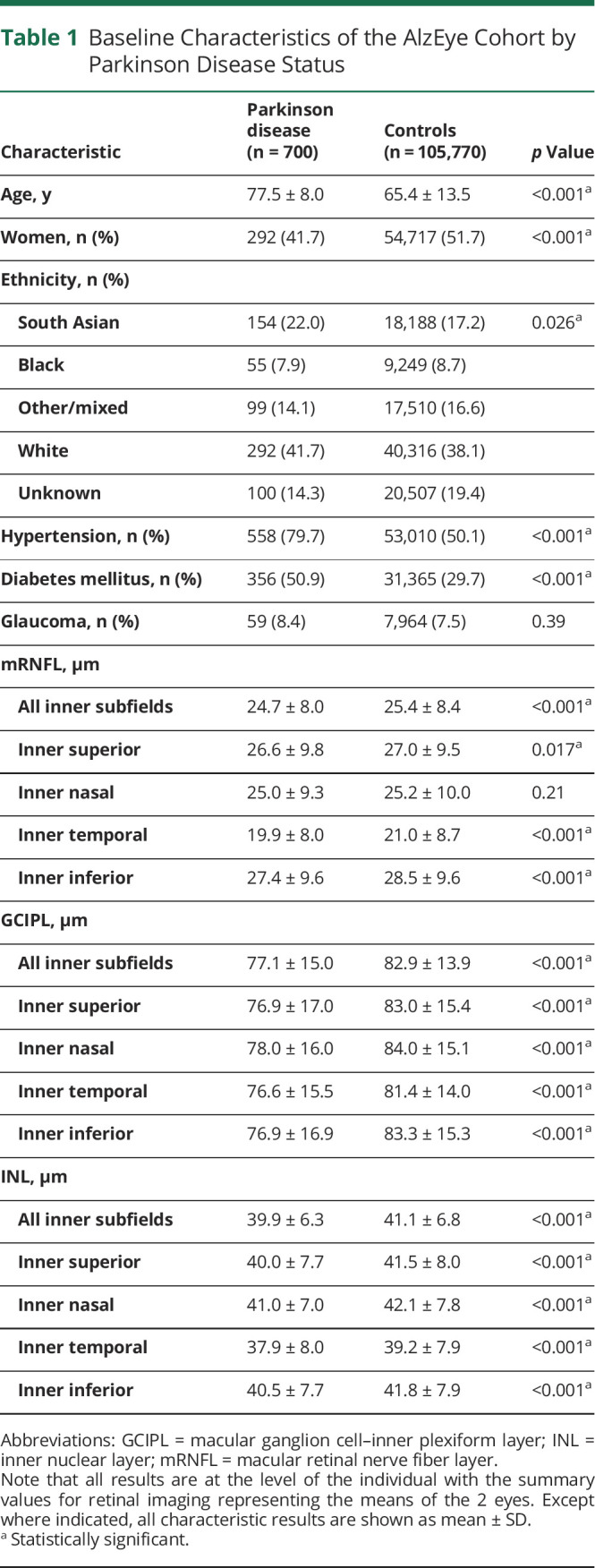
Baseline Characteristics of the AlzEye Cohort by Parkinson Disease Status

Characteristic	Parkinson disease (n = 700)	Controls (n = 105,770)	*p* Value
Age, y	77.5 ± 8.0	65.4 ± 13.5	<0.001^a^
Women, n (%)	292 (41.7)	54,717 (51.7)	<0.001^a^
Ethnicity, n (%)			
South Asian	154 (22.0)	18,188 (17.2)	0.026^a^
Black	55 (7.9)	9,249 (8.7)	
Other/mixed	99 (14.1)	17,510 (16.6)	
White	292 (41.7)	40,316 (38.1)	
Unknown	100 (14.3)	20,507 (19.4)	
Hypertension, n (%)	558 (79.7)	53,010 (50.1)	<0.001^a^
Diabetes mellitus, n (%)	356 (50.9)	31,365 (29.7)	<0.001^a^
Glaucoma, n (%)	59 (8.4)	7,964 (7.5)	0.39
mRNFL, μm			
All inner subfields	24.7 ± 8.0	25.4 ± 8.4	<0.001^a^
Inner superior	26.6 ± 9.8	27.0 ± 9.5	0.017^a^
Inner nasal	25.0 ± 9.3	25.2 ± 10.0	0.21
Inner temporal	19.9 ± 8.0	21.0 ± 8.7	<0.001^a^
Inner inferior	27.4 ± 9.6	28.5 ± 9.6	<0.001^a^
GCIPL, μm			
All inner subfields	77.1 ± 15.0	82.9 ± 13.9	<0.001^a^
Inner superior	76.9 ± 17.0	83.0 ± 15.4	<0.001^a^
Inner nasal	78.0 ± 16.0	84.0 ± 15.1	<0.001^a^
Inner temporal	76.6 ± 15.5	81.4 ± 14.0	<0.001^a^
Inner inferior	76.9 ± 16.9	83.3 ± 15.3	<0.001^a^
INL, μm			
All inner subfields	39.9 ± 6.3	41.1 ± 6.8	<0.001^a^
Inner superior	40.0 ± 7.7	41.5 ± 8.0	<0.001^a^
Inner nasal	41.0 ± 7.0	42.1 ± 7.8	<0.001^a^
Inner temporal	37.9 ± 8.0	39.2 ± 7.9	<0.001^a^
Inner inferior	40.5 ± 7.7	41.8 ± 7.9	<0.001^a^

Abbreviations: GCIPL = macular ganglion cell–inner plexiform layer; INL = inner nuclear layer; mRNFL = macular retinal nerve fiber layer.

Note that all results are at the level of the individual with the summary values for retinal imaging representing the means of the 2 eyes. Except where indicated, all characteristic results are shown as mean ± SD.

a Statistically significant.

After adjustment for age, sex, ethnicity, hypertension, and diabetes mellitus, individuals with prevalent PD had significantly thinner GCIPL across all parafoveal subfields (all inner −2.12 μm, 95% CI −3.17 to −1.07, *p* = 8.2 × 10^−5^). Thickness point estimates were most reduced in the inferior subfield (−2.38 μm, 95% CI −3.54 to −1.22, *p* = 6.0 × 10^−5^) and least reduced in the temporal subfield (−1.75 μm, 95% CI −2.82 to −0.68, *p* = 0.001). Individuals with PD also had significantly reduced thickness of the INL across all subfields (all inner −0.99 μm, 95% CI −1.52 to −0.47, *p* = 2.1 × 10^−4^), most marked at the superior subfield (−1.09 μm, 95% CI −1.70 to −0.47, *p* = 5.9 × 10^−4^). There was limited evidence of reduced mRNFL thickness and prevalent PD with only a weak association seen for the inner temporal subfield (−0.69 μm, 95% CI −1.37 to −0.02, *p* = 0.045, Table 2).

**Table 2:**
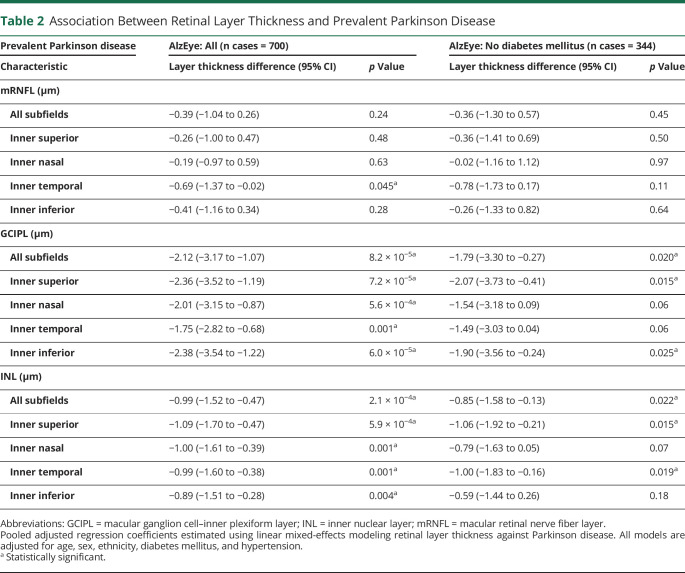
Association Between Retinal Layer Thickness and Prevalent Parkinson Disease

Prevalent Parkinson disease	AlzEye: All (n cases = 700)		AlzEye: No diabetes mellitus (n cases = 344)	
Characteristic	Layer thickness difference (95% CI)	*p* Value	Layer thickness difference (95% CI)	*p* Value
mRNFL (μm)				
All subfields	−0.39 (−1.04 to 0.26)	0.24	−0.36 (−1.30 to 0.57)	0.45
Inner superior	−0.26 (−1.00 to 0.47)	0.48	−0.36 (−1.41 to 0.69)	0.50
Inner nasal	−0.19 (−0.97 to 0.59)	0.63	−0.02 (−1.16 to 1.12)	0.97
Inner temporal	−0.69 (−1.37 to −0.02)	0.045^a^	−0.78 (−1.73 to 0.17)	0.11
Inner inferior	−0.41 (−1.16 to 0.34)	0.28	−0.26 (−1.33 to 0.82)	0.64
GCIPL (μm)				
All subfields	−2.12 (−3.17 to −1.07)	8.2 × 10^−5a^	−1.79 (−3.30 to −0.27)	0.020^a^
Inner superior	−2.36 (−3.52 to −1.19)	7.2 × 10^−5a^	−2.07 (−3.73 to −0.41)	0.015^a^
Inner nasal	−2.01 (−3.15 to −0.87)	5.6 × 10^−4a^	−1.54 (−3.18 to 0.09)	0.06
Inner temporal	−1.75 (−2.82 to −0.68)	0.001^a^	−1.49 (−3.03 to 0.04)	0.06
Inner inferior	−2.38 (−3.54 to −1.22)	6.0 × 10^−5a^	−1.90 (−3.56 to −0.24)	0.025^a^
INL (μm)				
All subfields	−0.99 (−1.52 to −0.47)	2.1 × 10^−4a^	−0.85 (−1.58 to −0.13)	0.022^a^
Inner superior	−1.09 (−1.70 to −0.47)	5.9 × 10^−4a^	−1.06 (−1.92 to −0.21)	0.015^a^
Inner nasal	−1.00 (−1.61 to −0.39)	0.001^a^	−0.79 (−1.63 to 0.05)	0.07
Inner temporal	−0.99 (−1.60 to −0.38)	0.001^a^	−1.00 (−1.83 to −0.16)	0.019^a^
Inner inferior	−0.89 (−1.51 to −0.28)	0.004^a^	−0.59 (−1.44 to 0.26)	0.18

Abbreviations: GCIPL = macular ganglion cell–inner plexiform layer; INL = inner nuclear layer; mRNFL = macular retinal nerve fiber layer.

Pooled adjusted regression coefficients estimated using linear mixed-effects modeling retinal layer thickness against Parkinson disease. All models are adjusted for age, sex, ethnicity, diabetes mellitus, and hypertension.

a Statistically significant.

Exclusion of all individuals with diabetes mellitus left a cohort of 344 individuals with PD and 74,405 controls. Effect measures were slightly reduced, but significant associations were still seen between PD and the thickness of both the GCIPL (all inner −1.79 μm, 95% CI −3.30 to −0.27, *p* = 0.020) and the INL (all inner −0.85 μm, 95% CI −1.58 to −0.13, *p* = 0.022). There were no associations between mRNFL and prevalent PD in this restricted group (all inner −0.36 μm, 95% CI −1.30 to 0.57, *p* = 0.45).

### Retinal Markers and Incident PD

**Figure 3:**
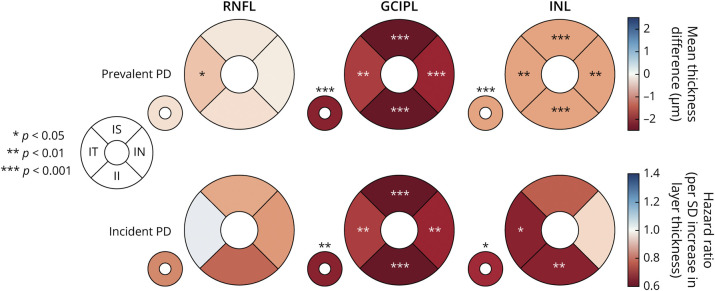
Summary of Findings for Prevalent and Incident Parkinson Disease Values are shown per parafoveal region and for the average of all inner segments (small donut). The effect measure corresponds to a color scale with warm colors indicating lower numbers. GCIPL = macular ganglion cell–inner plexiform layer; II = inner inferior; IN = inner nasal; INL = inner nuclear layer; IS = inner superior; IT = inner temporal; mRNFL = macular retinal nerve fiber layer.

From 67,311 participants in UKBB who underwent extended ophthalmic assessment as part of their baseline visit, 50,405 individuals had images of sufficient quality for analysis and fit the inclusion criteria (Figure 1). The cohort had a mean age of 56.1 ± 8.2 years, 54.7% were women, and people were predominantly of White self-reported ethnicity (91.4%). Fifty-three individuals developed PD during the study period (eTable 4, links.lww.com/WNL/D56). Among those with incident PD, the average time between retinal imaging and clinical presentation was 2,653 ± 851 days. On adjusted survival analysis, age and male sex were significantly associated with incident PD (Table 3). Regarding retinal markers, reduced thickness of the GCIPL was associated with incident PD (HR 0.62, 95% CI 0.46–0.84 per SD increase, *p* = 0.002, Figure 3). There was also some evidence that thinner INL was associated with incident PD, especially at the inferior subfield (HR 0.66, 95% CI 0.51–0.86, *p* = 0.002). The key findings of thinner GCIPL and INL being associated with greater likelihood of developing PD persisted even when all those who developed PD in the first 24 months after having had retinal imaging were excluded (Table 4).

**Table 3:**
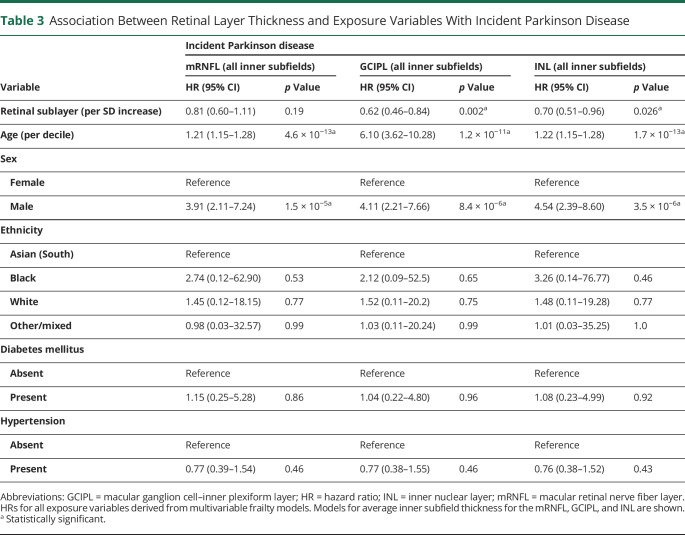
Association Between Retinal Layer Thickness and Exposure Variables With Incident Parkinson Disease

Variable	Incident Parkinson disease					
	mRNFL (all inner subfields)		GCIPL (all inner subfields)		INL (all inner subfields)	
	HR (95% CI)	*p* Value	HR (95% CI)	*p* Value	HR (95% CI)	*p* Value
Retinal sublayer (per SD increase)	0.81 (0.60–1.11)	0.19	0.62 (0.46–0.84)	0.002^a^	0.70 (0.51–0.96)	0.026^a^
Age (per decile)	1.21 (1.15–1.28)	4.6 × 10^−13a^	6.10 (3.62–10.28)	1.2 × 10^−11a^	1.22 (1.15–1.28)	1.7 × 10^−13a^
Sex						
Female	Reference		Reference		Reference	
Male	3.91 (2.11–7.24)	1.5 × 10^−5a^	4.11 (2.21–7.66)	8.4 × 10^−6a^	4.54 (2.39–8.60)	3.5 × 10^−6a^
Ethnicity						
Asian (South)	Reference		Reference		Reference	
Black	2.74 (0.12–62.90)	0.53	2.12 (0.09–52.5)	0.65	3.26 (0.14–76.77)	0.46
White	1.45 (0.12–18.15)	0.77	1.52 (0.11–20.2)	0.75	1.48 (0.11–19.28)	0.77
Other/mixed	0.98 (0.03–32.57)	0.99	1.03 (0.11–20.24)	0.99	1.01 (0.03–35.25)	1.0
Diabetes mellitus						
Absent	Reference		Reference		Reference	
Present	1.15 (0.25–5.28)	0.86	1.04 (0.22–4.80)	0.96	1.08 (0.23–4.99)	0.92
Hypertension						
Absent	Reference		Reference		Reference	
Present	0.77 (0.39–1.54)	0.46	0.77 (0.38–1.55)	0.46	0.76 (0.38–1.52)	0.43

Abbreviations: GCIPL = macular ganglion cell–inner plexiform layer; HR = hazard ratio; INL = inner nuclear layer; mRNFL = macular retinal nerve fiber layer.

HRs for all exposure variables derived from multivariable frailty models. Models for average inner subfield thickness for the mRNFL, GCIPL, and INL are shown.

a Statistically significant.

**Table 4:**
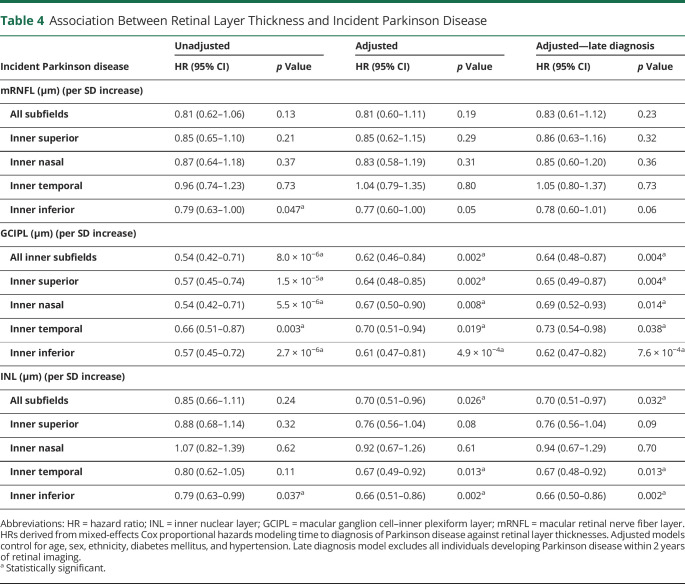
Association Between Retinal Layer Thickness and Incident Parkinson Disease

Incident Parkinson disease	Unadjusted		Adjusted		Adjusted—late diagnosis	
	HR (95% CI)	*p* Value	HR (95% CI)	*p* Value	HR (95% CI)	*p* Value
mRNFL (μm) (per SD increase)						
All subfields	0.81 (0.62–1.06)	0.13	0.81 (0.60–1.11)	0.19	0.83 (0.61–1.12)	0.23
Inner superior	0.85 (0.65–1.10)	0.21	0.85 (0.62–1.15)	0.29	0.86 (0.63–1.16)	0.32
Inner nasal	0.87 (0.64–1.18)	0.37	0.83 (0.58–1.19)	0.31	0.85 (0.60–1.20)	0.36
Inner temporal	0.96 (0.74–1.23)	0.73	1.04 (0.79–1.35)	0.80	1.05 (0.80–1.37)	0.73
Inner inferior	0.79 (0.63–1.00)	0.047^a^	0.77 (0.60–1.00)	0.05	0.78 (0.60–1.01)	0.06
GCIPL (μm) (per SD increase)						
All inner subfields	0.54 (0.42–0.71)	8.0 × 10^−6a^	0.62 (0.46–0.84)	0.002^a^	0.64 (0.48–0.87)	0.004^a^
Inner superior	0.57 (0.45–0.74)	1.5 × 10^−5a^	0.64 (0.48–0.85)	0.002^a^	0.65 (0.49–0.87)	0.004^a^
Inner nasal	0.54 (0.42–0.71)	5.5 × 10^−6a^	0.67 (0.50–0.90)	0.008^a^	0.69 (0.52–0.93)	0.014^a^
Inner temporal	0.66 (0.51–0.87)	0.003^a^	0.70 (0.51–0.94)	0.019^a^	0.73 (0.54–0.98)	0.038^a^
Inner inferior	0.57 (0.45–0.72)	2.7 × 10^−6a^	0.61 (0.47–0.81)	4.9 × 10^−4a^	0.62 (0.47–0.82)	7.6 × 10^−4a^
INL (μm) (per SD increase)						
All subfields	0.85 (0.66–1.11)	0.24	0.70 (0.51–0.96)	0.026^a^	0.70 (0.51–0.97)	0.032^a^
Inner superior	0.88 (0.68–1.14)	0.32	0.76 (0.56–1.04)	0.08	0.76 (0.56–1.04)	0.09
Inner nasal	1.07 (0.82–1.39)	0.62	0.92 (0.67–1.26)	0.61	0.94 (0.67–1.29)	0.70
Inner temporal	0.80 (0.62–1.05)	0.11	0.67 (0.49–0.92)	0.013^a^	0.67 (0.48–0.92)	0.013^a^
Inner inferior	0.79 (0.63–0.99)	0.037^a^	0.66 (0.51–0.86)	0.002^a^	0.66 (0.50–0.86)	0.002^a^

Abbreviations: HR = hazard ratio; INL = inner nuclear layer; GCIPL = macular ganglion cell–inner plexiform layer; mRNFL = macular retinal nerve fiber layer.

HRs derived from mixed-effects Cox proportional hazards modeling time to diagnosis of Parkinson disease against retinal layer thicknesses. Adjusted models control for age, sex, ethnicity, diabetes mellitus, and hypertension. Late diagnosis model excludes all individuals developing Parkinson disease within 2 years of retinal imaging.

a Statistically significant.

## Discussion

In this cross-sectional analysis of the AlzEye and UKBB cohorts, we first confirm earlier reports that individuals with PD have significantly thinner GCIPL. Second, prevalent PD is also associated with thinner INL, which is a novel finding. This is relevant because the INL represents the hub of dopaminergic activity in the neurosensory retina. Third, we found evidence that reduced thickness of the GCIPL and, to some extent, the INL is also associated with an increased chance of developing PD beyond that which is conferred by age, sex, ethnicity, hypertension, and diabetes mellitus. Collectively, these findings strengthen the argument that neurodegenerative pathology in PD involves the GCIPL and INL and that these retinal layers may have prognostic clinical relevance.

**Figure 4:**
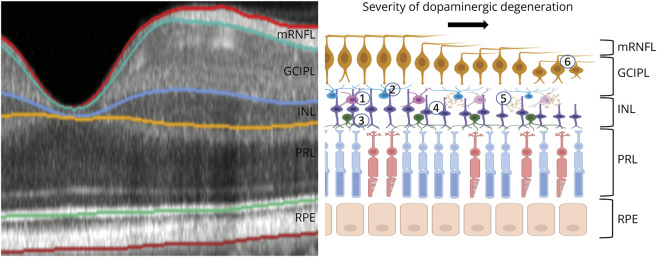
Illustration of Cell Type Distribution in the Retina An example optical coherence tomography scan of the nasal macula adjacent to schematic detailing interactions with dopaminergic amacrine cells. Dopaminergic cells (1) have dense plexi positioned throughout the inner plexiform and inner nuclear layers. They are presynaptic to amacrine AII cells (2), and some dopaminergic processes project toward the photoreceptor layer where they interact with horizontal cells (3). They are postsynaptic to bipolar cells (4). Previous work has demonstrated aggregation of proteins, including α synuclein, within the inner nuclear layer (5), which could result in impairment of nearby ganglion cells. GCIPL = macular ganglion cell–inner plexiform layer; INL = inner nuclear layer; PRL = photoreceptor layer; RNFL = retinal nerve fiber layer; RPE = retinal pigment epithelium.

The INL acts as a barrier to propagation of retrograde trans-synaptic axonal degeneration (RTSD) from the brain to the eye.^29^ The anatomical reason for this is the network function of the INL which involves horizontal connections of bipolar cells with amacrine and horizontal cells. Data on the preservation of INL thickness have been confirmed in the second of 2 large meta-analyses in multiple sclerosis.^8,30^ In contrast to the INL, there is atrophy of the peripapillary RNFL which is robust on repeat meta-analyses over 2 decades and different OCT devices. Therefore, the finding of reduced INL thickness in the present cohort is not only novel but also permits formulation of a new hypothesis of retinal neurodegeneration in PD (Figure 4). Although the effect measure was modest (∼1 μm), we found reduced INL thickness consistently across all parafoveal segments in prevalent PD and in the inferior and temporal subfields in incident PD. Studies thus far have likely been underpowered to detect this new effect. In a 2021 meta-analysis of a total of 387 participants across 4 reports, only 2 showed significant associations between INL thickness and prevalent PD but with opposite directions of effect.^11^ While Schneider et al.^31^ found a reduction in INL thickness (mean 1.2 μm) when comparing 65 patients with PD against age and sex-matched controls, Albrecht et al.^32^ noted a mean increase of 4 μm. Participants in the latter work were younger on average (61.2 ± 2.0 vs 66.2 ± 12), but both had similar disease duration and severity. Dopaminergic activity in the inner retina predominantly comes from the amacrine cells,^6^ which interface with retinal ganglion and AII amacrine cells. Intracellular alpha-synuclein aggregates have been found in the INL^33^ and individuals with PD have significantly reduced dopaminergic amacrine cells in the retina on immunohistochemistry.^34^ Inner retinal accumulation of toxic protein aggregates provide a plausible explanation for reduced INL thickness (Figure 4). On a molecular level, toxic aggregates lead to increased free radicals, oxidative stress, and mitochondrial damage ultimately leading to energy deficiency and neurodegeneration. Thus, a biologically plausible explanation for our finding could be a primary inner retinal PD-related dopaminergic degeneration manifesting as INL thinning on OCT. This explanation reconciles our data on reduced INL thickness in PD with the general absence of INL atrophy in nondopaminergic neurologic disorders, where inner retinal change arises from RTSD.^8^

We found individuals with PD had significantly thinner GCIPL, most prominent at the superior and inferior subfields and persisting when excluding all patients with diabetes. For context, our effect estimate (−2.12 μm) equates to approximately 14 years in a recent UKBB cohort analysis.^35^ Across 690 participants in 10 studies, Huang et al. found that people with PD had on average 3.17 μm (95% CI −5.07 to −1.26) thinner GCIPL compared with controls, with the inferior subfield exhibiting the greatest difference (−7.86 μm). GCIPL thinning has similarly been reported in Alzheimer disease and after ischemic stroke mediated through RTSD.^36,37^ Even among neurologically healthy older individuals, a thinner GCIPL is associated with gray matter volume and brain atrophy.^38^ Gray matter atrophy is found in patients with PD, but it is heterogenous and inconsistent,^39^ possibly because gray matter atrophy represents neuronal cell death^40^ which is a relatively late event in PD. Instead, animal models suggest that axonal changes are likely to be earlier events,^41^ and this is supported by degeneration of white matter brain connections before cortical atrophy in PD.^42^ In the retina, dopaminergic cell bodies are found at the border of the INL and IPL, with axons projecting along the GCIPL. We can consider 2 potential mechanistic explanations for the reduced GCIPL thickness we have observed in PD. First, cerebral neurodegeneration in PD may induce GCIPL thinning through RTSD given similar mechanisms seen in other neurodegenerative diseases. An alternative possibility is a local effect originating with dopaminergic dysfunction in the INL or in situ axonal degeneration of the retinal ganglion cell. Although dopaminergic neurons represent <1% of all amacrine cells in the INL (density of 10–100/mm^2^),^43^ they reach their peak density in the parafoveal region in healthy primates (corresponding to the 1–3 mm ETDRS area investigated in this report).^34^ Moreover, retinal dopaminergic dysfunction in humans with PD has previously been linked with death of adjacent cells, particularly ganglion cells. The dopaminergic amacrine cells couple to melanopsin-sensitive retinal ganglion cells in the GCIPL, and immunohistochemistry shows that reduced dopaminergic plexi in individuals with PD are accompanied by abnormal retinal ganglion cell morphology.^34^ Immunohistochemical staining for dopamine was almost absent from the INL in PD. Therefore, while the GCIPL and INL atrophy observed in the parafoveal region may predominantly involve other cell types, it is likely to be pathophysiologically related to dopaminergic cell death or dysfunction. Future studies are needed to determine whether progression of GCIPL atrophy in PD is driven by retrograde mechanism from the posterior thalamus (e.g., lateral geniculate nucleus atrophy precedes GCIPL atrophy) or anterograde from the INL (INL thinning precedes GCIPL atrophy).

In our report, thinner INL and GCIPL were also associated with a higher risk of developing PD. However, it should be noted that the effect sizes, especially for the INL, are small, so the practical value for an individual as a marker of early PD is currently limited. The association between retinal layer thicknesses and incident PD had not yet been explored; however, findings in early and prodromal PD do corroborate our results. Reduced thickness of the GCIPL has been described in individuals with drug-naive PD and is related to severity of disease.^44,45^ Individuals with idiopathic REM sleep behavior disorder, a variant of prodromal PD where >70% of affected individuals may convert to a Lewy body disease,^46^ have thinner ganglion cell complexes on OCT with the severity related to the degree of nigrostriatal dopaminergic degeneration.^47^ Epidemiologic patterns in other neurodegenerative diseases have also suggested inner retinal changes may occur early. In the Rotterdam Study, Mutlu et al.^19^ found that individuals with thinner mRNFL on OCT had an increased risk of developing Alzheimer dementia. Another neurodegenerative explanation for the reduced inner retinal thickness observed could be glaucomatous optic neuropathy. The association between glaucoma and PD is conflicting, and a recent meta-analysis concluded that glaucoma was not associated with an increased risk of PD.^48^ In our AlzEye cohort, the prevalence of glaucoma was relatively similar in those with PD (8.4%) and controls (7.5%) despite the group with PD being 12 years older on average. For the UKBB analysis, we excluded all individuals with previously diagnosed glaucoma; however, it is conceivable that individuals at risk of PD may have either undiagnosed and/or early-stage glaucoma. Ophthalmic deep phenotyping in, for example, prodromal PD would help identify the interplay between development of glaucoma and progression to a synucleinopathy.

Our study has limitations. First, for our prevalent PD analysis, we did not have detailed clinical information about PD status, such as diagnosis date, treatment patterns, or current therapy. We were therefore not able to relate retinal morphology to disease duration or severity, although retinal thicknesses have not been shown to differ between individuals with treated and untreated PD.^49^ Second, our case definition of PD was based on *ICD-10* codes rather than a PD-specific reference standard. *ICD-10* codes from HES for PD have been validated in a subset of 20,000 UKBB participants and shown to have a positive predictive value of 0.84 (0.68–0.94).^18^ A separate report at a large tertiary NHS hospital showed 27% of hospital admissions of individuals with PD did not have PD recorded (i.e., sensitivity of 0.73).^50^ Thus, our effect sizes are likely to be biased toward the null because controls may in fact have PD. Finally, we do not have correlative OCT and retinal histology data on the proposed protein aggregation hypothesis in the INL. Such data will depend on tissue donation for research purposes by individuals with PD who have had in vivo OCT, such as the UK PD Brain Bank.

In conclusion, our report demonstrates that individuals with PD have significantly thinner GCIPL and the INL. These differences appear early, being discernible several years before clinical presentation. It remains unclear whether such changes relate to the increased neurodegeneration found in the brains of individuals with PD and resulting RTSD or could represent a primary dopaminergic degeneration focused within the inner retina with anterior propagation of neurodegeneration. Further studies exploring the chronological sequence of retinal sublayer thickness would help elucidate the mechanism and determine whether retinal imaging could support the diagnosis, prognosis, and complex management of patients affected by PD.

## Appendix 1 Authors

**Table:**

Name	Location	Contribution
Siegfried Karl Wagner, MSc, MD	Institute of Ophthalmology, University College London; NIHR Biomedical Research Centre at Moorfields Eye Hospital and UCL Institute of Ophthalmology, London, United Kingdom	Drafting/revision of the manuscript for content, including medical writing for content; major role in the acquisition of data; study concept or design; analysis or interpretation of data
David Romero-Bascones, BSc	NIHR Biomedical Research Centre at Moorfields Eye Hospital and UCL Institute of Ophthalmology, London, United Kingdom; Biomedical Engineering Department, Faculty of Engineering (MU-ENG), Mondragon Unibertsitatea, Spain	Drafting/revision of the manuscript for content, including medical writing for content; major role in the acquisition of data; study concept or design; analysis or interpretation of data
Mario Cortina-Borja, PhD	Great Ormond Street Institute of Child Health, University College London, United Kingdom	Drafting/revision of the manuscript for content, including medical writing for content; major role in the acquisition of data; study concept or design; analysis or interpretation of data
Dominic J. Williamson, MSc	Institute of Ophthalmology, University College London; NIHR Biomedical Research Centre at Moorfields Eye Hospital and UCL Institute of Ophthalmology; Centre for Medical Image Computing, Department of Computer Science, University College London, United Kingdom	Drafting/revision of the manuscript for content, including medical writing for content; major role in the acquisition of data; study concept or design; analysis or interpretation of data
Robbert R. Struyven, MSc	Institute of Ophthalmology, University College London; NIHR Biomedical Research Centre at Moorfields Eye Hospital and UCL Institute of Ophthalmology; Centre for Medical Image Computing, Department of Computer Science, University College London, United Kingdom	Drafting/revision of the manuscript for content, including medical writing for content; major role in the acquisition of data; study concept or design; analysis or interpretation of data
Yukun Zhou, MSc	Institute of Ophthalmology, University College London; NIHR Biomedical Research Centre at Moorfields Eye Hospital and UCL Institute of Ophthalmology; Centre for Medical Image Computing, Department of Computer Science, University College London, United Kingdom	Drafting/revision of the manuscript for content, including medical writing for content; major role in the acquisition of data; study concept or design; analysis or interpretation of data
Salil Patel, MD	NeuroMetrology Lab, Nuffield Department of Clinical Neurosciences, University of Oxford, United Kingdom	Drafting/revision of the manuscript for content, including medical writing for content; analysis or interpretation of data
Rimona S. Weil, PhD	Dementia Research Centre, University College London, United Kingdom	Drafting/revision of the manuscript for content, including medical writing for content; analysis or interpretation of data
Chrystalina A. Antoniades, PhD	NeuroMetrology Lab, Nuffield Department of Clinical Neurosciences, University of Oxford, United Kingdom	Drafting/revision of the manuscript for content, including medical writing for content; analysis or interpretation of data
Eric J. Topol, MD	Department of Molecular Medicine, Scripps Research, La Jolla, CA	Drafting/revision of the manuscript for content, including medical writing for content; analysis or interpretation of data
Edward Korot, MD	NIHR Biomedical Research Centre at Moorfields Eye Hospital and UCL Institute of Ophthalmology, London, United Kingdom; Biomedical Engineering Department, Faculty of Engineering (MU-ENG), Mondragon Unibertsitatea, Spain; Byers Eye Institute, Stanford University, Palo Alto, CA	Drafting/revision of the manuscript for content, including medical writing for content; analysis or interpretation of data
Paul J. Foster, PhD	Institute of Ophthalmology, University College London; NIHR Biomedical Research Centre at Moorfields Eye Hospital and UCL Institute of Ophthalmology, London, United Kingdom	Drafting/revision of the manuscript for content, including medical writing for content; analysis or interpretation of data
Konstantinos Balaskas, MD	Institute of Ophthalmology, University College London; NIHR Biomedical Research Centre at Moorfields Eye Hospital and UCL Institute of Ophthalmology, London, United Kingdom	Drafting/revision of the manuscript for content, including medical writing for content; analysis or interpretation of data
Unai Ayala, PhD	Biomedical Engineering Department, Faculty of Engineering (MU-ENG), Mondragon Unibertsitatea, Spain	Drafting/revision of the manuscript for content, including medical writing for content; analysis or interpretation of data
Maitane Barrenechea, PhD	Biomedical Engineering Department, Faculty of Engineering (MU-ENG), Mondragon Unibertsitatea, Spain	Drafting/revision of the manuscript for content, including medical writing for content; analysis or interpretation of data
Iñigo Gabilondo, MD, PhD	Biocruces Bizkaia Health Research Institute, Barakaldo; IKERBASQUE: The Basque Foundation for Science, Bilbao, Spain	Drafting/revision of the manuscript for content, including medical writing for content; analysis or interpretation of data
Anthony H.V. Schapira, MD	Department of Clinical and Movement Neurosciences, UCL Queen Square Institute of Neurology, London, United Kingdom	Drafting/revision of the manuscript for content, including medical writing for content; analysis or interpretation of data
Anthony P. Khawaja, PhD	Institute of Ophthalmology, University College London; NIHR Biomedical Research Centre at Moorfields Eye Hospital and UCL Institute of Ophthalmology, London, United Kingdom	Drafting/revision of the manuscript for content, including medical writing for content; analysis or interpretation of data
Praveen J. Patel, MD	Institute of Ophthalmology, University College London; NIHR Biomedical Research Centre at Moorfields Eye Hospital and UCL Institute of Ophthalmology, London, United Kingdom	Drafting/revision of the manuscript for content, including medical writing for content; analysis or interpretation of data
Jugnoo S. Rahi, PhD	Institute of Ophthalmology, University College London; NIHR Biomedical Research Centre at Moorfields Eye Hospital and UCL Institute of Ophthalmology; Great Ormond Street Institute of Child Health, University College London; Great Ormond Street Hospital NHS Foundation Trust; Ulverscroft Vision Research Group, University College London, United Kingdom	Drafting/revision of the manuscript for content, including medical writing for content; major role in the acquisition of data; study concept or design; analysis or interpretation of data
Alastair K. Denniston, PhD	NIHR Biomedical Research Centre at Moorfields Eye Hospital and UCL Institute of Ophthalmology, London; University of Birmingham; University Hospitals Birmingham NHS Foundation Trust; NIHR Birmingham Biomedical Research Centre, University of Birmingham, United Kingdom	Drafting/revision of the manuscript for content, including medical writing for content; major role in the acquisition of data; study concept or design; analysis or interpretation of data
Axel Petzold, MD, PhD	Institute of Ophthalmology, University College London; NIHR Biomedical Research Centre at Moorfields Eye Hospital and UCL Institute of Ophthalmology, London; Queen Square Institute of Neurology, University College London, United Kingdom	Drafting/revision of the manuscript for content, including medical writing for content; major role in the acquisition of data; study concept or design; analysis or interpretation of data
Pearse Andrew Keane, MD	Institute of Ophthalmology, University College London; NIHR Biomedical Research Centre at Moorfields Eye Hospital and UCL Institute of Ophthalmology, London, United Kingdom	Drafting/revision of the manuscript for content, including medical writing for content; major role in the acquisition of data; study concept or design; analysis or interpretation of data

## Appendix 2 Coinvestigators

**Table:**

Coinvestigators are listed at links.lww.com/WNL/D57.

## Glossary

DAT: dopamine transport
ETDRS: Early Treatment for Diabetic Retinopathy Study
GCIPL: macular ganglion cell–inner plexiform layer
HES: Hospital Episode Statistics
HR: hazard ratio
*ICD-10*: *International Classification of Diseases, 10th Revision*
INL: inner nuclear layer
IPL: inner plexiform layer
mRNFL: macular retinal nerve fiber layer
OCT: optical coherence tomography
PD: Parkinson disease
RTSD: retrograde trans-synaptic axonal degeneration
TABS: Topcon Advanced Boundary Segmentation Tool
UKBB: UK Biobank

## References

[1] Biondetti E, Santin MD, Valabrègue R, et al. The spatiotemporal changes in dopamine, neuromelanin and iron characterizing Parkinson's disease. Brain. 2021;144(10):3114-3125. doi:10.1093/brain/awab191 33978742 PMC8634084

[2] Morrish PK, Sawle GV, Brooks DJ. An [18F]dopa-PET and clinical study of the rate of progression in Parkinson's disease. Brain. 1996;119(2):585-591. doi:10.1093/brain/119.2.585 8800950

[3] Sun J, Lai Z, Ma J, et al. Quantitative evaluation of iron content in idiopathic rapid eye movement sleep behavior disorder. Mov Disord. 2020;35(3):478-485. doi:10.1002/mds.27929 31846123

[4] Iranzo A, Valldeoriola F, Lomeña F, et al. Serial dopamine transporter imaging of nigrostriatal function in patients with idiopathic rapid-eye-movement sleep behaviour disorder: a prospective study. Lancet Neurol. 2011;10(9):797-805. doi:10.1016/s1474-4422(11)70152-1 21802993

[5] Lee JY, Martin-Bastida A, Murueta-Goyena A, et al. Multimodal brain and retinal imaging of dopaminergic degeneration in Parkinson disease. Nat Rev Neurol. 2022;18(4):203-220. doi:10.1038/s41582-022-00618-9 35177849

[6] Witkovsky P. Dopamine and retinal function. Doc Ophthalmol. 2004;108(1):17-40. doi:10.1023/b:doop.0000019487.88486.0a 15104164

[7] Harnois C, Di Paolo T. Decreased dopamine in the retinas of patients with Parkinson's disease. Invest Ophthalmol Vis Sci. 1990;31(11):2473-2475. 2243012

[8] Petzold A, Balcer LJ, Calabresi PA, et al. Retinal layer segmentation in multiple sclerosis: a systematic review and meta-analysis. Lancet Neurol. 2017;16(10):797-812. doi:10.1016/s1474-4422(17)30278-8 28920886

[9] Vijay V, Mollan SP, Mitchell JL, et al. Using optical coherence tomography as a surrogate of measurements of intracranial pressure in idiopathic intracranial hypertension. JAMA Ophthalmol. 2020;138(12):1264-1271. doi:10.1001/jamaophthalmol.2020.4242 33090189 PMC7582233

[10] Kiely PM, Cappuccio S, McIntyre E. Optometry Australia Scope of Practice Survey 2015. Clin Exp Optom. 2017;100(3):260-269. doi:10.1111/cxo.12538 28295595

[11] Huang L, Zhang D, Ji J, Wang Y, Zhang R. Central retina changes in Parkinson's disease: a systematic review and meta-analysis. J Neurol. 2021;268(12):4646-4654. doi:10.1007/s00415-020-10304-9 33174132

[12] Dacey DM. The dopaminergic amacrine cell. J Comp Neurol. 1990;301(3):461-489. doi:10.1002/cne.903010310 1979792

[13] Komici K, Femminella GD, Bencivenga L, Rengo G, Pagano G. Diabetes mellitus and Parkinson's disease: a systematic review and meta-analyses. J Parkinsons Dis. 2021;11(4):1585-1596. doi:10.3233/jpd-212725 34486987

[14] Wagner SK, Hughes F, Cortina-Borja M, et al. AlzEye: longitudinal record-level linkage of ophthalmic imaging and hospital admissions of 353 157 patients in London, UK. BMJ Open. 2022;12(3):e058552. doi:10.1136/bmjopen-2021-058552 PMC8928293 35296488

[15] Keane PA, Grossi CM, Foster PJ, et al. Optical coherence tomography in the UK Biobank study: rapid automated analysis of retinal thickness for large population-based studies. PLoS One. 2016;11(10):e0164095. doi:10.1371/journal.pone.0164095 27716837 PMC5055325

[16] Photocoagulation for diabetic macular edema. Early Treatment Diabetic Retinopathy Study report number 1. Early Treatment Diabetic Retinopathy Study. Arch Ophthalmol. 1985;103(12):1796-1806. 2866759

[17] Healthcare Across the UK: A Comparison of the NHS in England, Scotland, Wales and Northern Ireland—National Audit Office (NAO) Report [online]. National Audit Office; 2012. Accessed August 2, 2022. nao.org.uk/report/healthcare-across-the-uk-a-comparison-of-the-nhs-in-england-scotland-wales-and-northern-ireland/.

[18] Website [online]. January 14, 2023. biobank.ctsu.ox.ac.uk/crystal/ukb/docs/alg_outcome_pdp.

[19] Mutlu U, Colijn JM, Ikram MA, et al. Association of retinal neurodegeneration on optical coherence tomography with dementia: a population-based study. JAMA Neurol. 2018;75(10):1256-1263. doi:10.1001/jamaneurol.2018.1563 29946702 PMC6233847

[20] Berrett TB, Samworth R. USP: An Independence Test That Improves on Pearson's Chi-Squared and the G-Test. Apollo - University of Cambridge Repository; 2021. January 14, 2023. repository.cam.ac.uk/handle/1810/330630. 10.1098/rspa.2021.0549 PMC8652272 35153605

[21] Satterthwaite FE. An approximate distribution of estimates of variance components. Biometrics. 1946;2(6):110-114. doi:10.2307/3002019 20287815

[22] Tsiampalis T, Panagiotakos DB. Missing-data analysis: socio-demographic, clinical and lifestyle determinants of low response rate on self-reported psychological and nutrition related multi-item instruments in the context of the ATTICA epidemiological study. BMC Med Res Methodol. 2020;20(1):148. doi:10.1186/s12874-020-01038-3 32513107 PMC7281925

[23] van Buuren S. Multiple imputation of discrete and continuous data by fully conditional specification. Stat Methods Med Res. 2007;16(3):219-242. doi:10.1177/0962280206074463 17621469

[24] Hens N, Wienke A, Aerts M, Molenberghs G. The correlated and shared gamma frailty model for bivariate current status data: an illustration for cross-sectional serological data. Stat Med. 2009;28(22):2785-2800. doi:10.1002/sim.3660 19591117

[25] Brown A, Kirichek O, Balkwill A, et al. Comparison of dementia recorded in routinely collected hospital admission data in England with dementia recorded in primary care. Emerg Themes Epidemiol. 2016;13(1):11. doi:10.1186/s12982-016-0053-z 27800007 PMC5084368

[26] von Elm E, Altman DG, Egger M, Pocock SJ, Gøtzsche PC, Vandenbroucke JP. The Strengthening the Reporting of Observational Studies in Epidemiology (STROBE) Statement: guidelines for reporting observational studies. Int J Surg. 2014;12:1495-1499. doi:10.1016/j.ijsu.2014.07.013 25046131

[27] Aytulun A, Cruz-Herranz A, Aktas O, et al. APOSTEL 2.0 recommendations for reporting quantitative optical coherence tomography studies. Neurology. 2021;97(2):68-79. doi:10.1212/wnl.0000000000012125 33910937 PMC8279566

[28] Cruz-Herranz A, Balk LJ, Oberwahrenbrock T, et al. The APOSTEL recommendations for reporting quantitative optical coherence tomography studies. Neurology. 2016;86(24):2303-2309. doi:10.1212/wnl.0000000000002774 27225223 PMC4909557

[29] Balk LJ, Steenwijk MD, Tewarie P, et al. Bidirectional trans-synaptic axonal degeneration in the visual pathway in multiple sclerosis. J Neurol Neurosurg Psychiatry. 2015;86(4):419-424. doi:10.1136/jnnp-2014-308189 24973342

[30] Petzold A, de Boer JF, Schippling S, et al. Optical coherence tomography in multiple sclerosis: a systematic review and meta-analysis. Lancet Neurol. 2010;9:921-932. doi:10.1016/s1474-4422(10)70168-x 20723847

[31] Schneider M, Müller HP, Lauda F, et al. Retinal single-layer analysis in Parkinsonian syndromes: an optical coherence tomography study. J Neural Transm. 2014;121(1):41-47. doi:10.1007/s00702-013-1072-3 23907408

[32] Albrecht P, Müller AK, Südmeyer M, et al. Optical coherence tomography in parkinsonian syndromes. PLoS One. 2012;7(4):e34891. doi:10.1371/journal.pone.0034891 22514688 PMC3325949

[33] Bodis-Wollner I, Kozlowski PB, Glazman S, Miri S. Α-synuclein in the inner retina in Parkinson disease. Ann Neurol. 2014;75(6):964-966. doi:10.1002/ana.24182 24816946

[34] Ortuño-Lizarán I, Sánchez-Sáez X, Lax P, et al. Dopaminergic retinal cell loss and visual dysfunction in Parkinson disease. Ann Neurol. 2020;88(5):893-906. doi:10.1002/ana.25897 32881029 PMC10005860

[35] Khawaja AP, Chua S, Hysi PG, et al. Comparison of associations with different macular inner retinal thickness parameters in a large cohort: the UK Biobank. Ophthalmology. 2020;127(1):62-71. doi:10.1016/j.ophtha.2019.08.015 31585827

[36] Jindahra P, Petrie A, Plant GT. The time course of retrograde trans-synaptic degeneration following occipital lobe damage in humans. Brain. 2012;135(2):534-541. doi:10.1093/brain/awr324 22300877

[37] Chan VTT, Sun Z, Tang S, et al. Spectral-domain OCT measurements in Alzheimer's disease: a systematic review and meta-analysis. Ophthalmology. 2019;126(4):497-510. doi:10.1016/j.ophtha.2018.08.009 30114417 PMC6424641

[38] Mejia-Vergara AJ, Karanjia R, Sadun AA. OCT parameters of the optic nerve head and the retina as surrogate markers of brain volume in a normal population, a pilot study. J Neurol Sci. 2021;420:117213. doi:10.1016/j.jns.2020.117213 33271374

[39] Weil RS, Hsu JK, Darby RR, Soussand L, Fox MD. Neuroimaging in Parkinson's disease dementia: connecting the dots. Brain Commun. 2019;1:fcz006. doi:10.1093/braincomms/fcz006 31608325 PMC6777517

[40] Rossor MN, Fox NC, Freeborough PA, Roques PK. Slowing the progression of Alzheimer disease: monitoring progression. Alzheimer Dis Assoc Disord. 1997;11(suppl 5):S6-S9. 9348422

[41] Chung CY, Koprich JB, Siddiqi H, Isacson O. Dynamic changes in presynaptic and axonal transport proteins combined with striatal neuroinflammation precede dopaminergic neuronal loss in a rat model of AAV alpha-synucleinopathy. J Neurosci. 2009;29(11):3365-3373. doi:10.1523/jneurosci.5427-08.2009 19295143 PMC2693917

[42] Zarkali A, McColgan P, Leyland LA, Lees AJ, Weil RS. Visual dysfunction predicts cognitive impairment and white matter degeneration in Parkinson's disease. Mov Disord. 2021;36(5):1191-1202. doi:10.1002/mds.28477 33421201 PMC8248368

[43] Popova E. Role of Dopamine in Retinal Function. University of Utah Health Sciences Center; 2020. 32931183

[44] Lee JY, Ahn J, Yoon EJ, Oh S, Kim YK, Jeon B. Macular ganglion-cell-complex layer thinning and optic nerve integrity in drug-naïve Parkinson's disease. J Neural Transm. 2019;126(12):1695-1699. doi:10.1007/s00702-019-02097-7 31630254

[45] Ahn J, Lee JY, Kim TW, et al. Retinal thinning associates with nigral dopaminergic loss in de novo Parkinson disease. Neurology. 2018;91(11):e1003-e1012. doi:10.1212/wnl.0000000000006157 30111550

[46] Postuma RB, Gagnon JF, Bertrand JA, Génier Marchand D, Montplaisir JY. Parkinson risk in idiopathic REM sleep behavior disorder: preparing for neuroprotective trials. Neurology. 2015;84(11):1104-1113. doi:10.1212/wnl.0000000000001364 25681454 PMC4371408

[47] Lee JY, Ahn J, Oh S, et al. Retina thickness as a marker of neurodegeneration in prodromal Lewy body disease. Mov Disord. 2020;35(2):349-354. doi:10.1002/mds.27914 31710400

[48] Zhao B, Cheung R, Malvankar-Mehta MS. Risk of Parkinson's disease in glaucoma patients: a systematic review and meta-analysis. Curr Med Res Opin. 2022;38(6):955-962. doi:10.1080/03007995.2022.2070377 35475495

[49] Sen A, Tugcu B, Coskun C, Ekinci C, Nacaroglu SA. Effects of levodopa on retina in Parkinson disease. Eur J Ophthalmol. 2014;24(1):114-119. doi:10.5301/ejo.5000338 23828323

[50] Muzerengi S, Rick C, Begaj I, et al. Coding accuracy for Parkinson's disease hospital admissions: implications for healthcare planning in the UK. Public Health. 2017;146:4-9. doi:10.1016/j.puhe.2016.12.024 28404473

